# Functional rehabilitation of upper limb apraxia in poststroke patients: study protocol for a randomized controlled trial

**DOI:** 10.1186/s13063-015-1034-1

**Published:** 2015-11-05

**Authors:** Jose Manuel Pérez-Mármol, Mª Carmen García-Ríos, Francisco J. Barrero-Hernandez, Guadalupe Molina-Torres, Ted Brown, María Encarnación Aguilar-Ferrándiz

**Affiliations:** Department of Physical Therapy, University of Granada (UGR), Granada, Spain; Hospital Clínico San Cecilio, Granada, Spain; Department of Nursing and Physical Therapy, University of Almeria (UAL), Almeria, Spain; Department of Occupational Therapy, School of Primary Health Care, Faculty of Medicine, Nursing and Health Sciences, Monash University, Melbourne, Australia; Fisioterapia, Universidad de Granada, Granada, Spain

**Keywords:** Apraxia, Stroke, Rehabilitation

## Abstract

**Background:**

Upper limb apraxia is a common disorder associated with stroke that can reduce patients’ independence levels in activities of daily living and increase levels of disability. Traditional rehabilitation programs designed to promote the recovery of upper limb function have mainly focused on restorative or compensatory approaches. However, no previous studies have been completed that evaluate a combined intervention method approach, where patients concurrently receive cognitive training and learn compensatory strategies for enhancing daily living activities.

**Methods/Design:**

This study will use a two-arm, assessor-blinded, parallel, randomized controlled trial design, involving 40 patients who present a left- or right-sided unilateral vascular lesion poststroke and a clinical diagnosis of upper limb apraxia. Participants will be randomized to either a combined functional rehabilitation or a traditional health education group. The experimental group will receive an 8-week combined functional program at home, including physical and occupational therapy focused on restorative and compensatory techniques for upper limb apraxia, 3 days per week in 30-min intervention periods. The control group will receive a conventional health education program once a month over 8 weeks, based on improving awareness of physical and functional limitations and facilitating the adaptation of patients to the home. Study outcomes will be assessed immediately postintervention and at the 2-month follow-up. The primary outcome measure will be basic activities of daily living skills as assessed with the Barthel Index. Secondary outcome measures will include the following: 1) the Lawton and Brody Instrumental Activities of Daily Living Scale, 2) the Observation and Scoring of ADL-Activities, 3) the De Renzi Test for Ideational Apraxia, 4) the De Renzi Test for Ideomotor Apraxia, 5) Recognition of Gestures, 6) the Test of Upper Limb Apraxia (TULIA), and 7) the Quality of Life Scale For Stroke (ECVI-38).

**Discussion:**

This trial is expected to clarify the effectiveness of a combined functional rehabilitation approach compared to a conservative intervention for improving upper limb movement and function in poststroke patients.

**Trial registration:**

Clinical Trial Gov number NCT02199093. The protocol registration was received 23 July 2014. Participant enrollment began on 1 May 2014. The trial is expected to be completed in March 2016.

**Electronic supplementary material:**

The online version of this article (doi:10.1186/s13063-015-1034-1) contains supplementary material, which is available to authorized users.

## Background

Apraxia is considered the inability to carry out learned skilled motor acts despite the motor and sensory systems being intact with respect to coordination, comprehension, and cooperation [1, 2]. Specifically, this disorder is viewed as any motor ability problem acquired in the absence of motor impairments, such as weakness, akinesia, loss of sensory input, abnormal tone or posture, or movement disorder [3], which occurs as the result of a neurological dysfunction [4].

Apraxic impairments are classified as higher motor deficits since they may manifest in the absence of primary sensory and motor deficits, disturbed communication or lack of motivation. As the disorder appears when patients are processing goal-directed actions [5, 6], it has been characterized as a reduction in the patient’s ability to voluntarily perform goal-directed movements [7].

Limb apraxia (LA) is a subtype of apraxia covering a wide spectrum of higher motor disorders caused by acquired brain disease or injury and affecting the performance of skilled learned movements carried out by the upper limbs [8]. It cannot be explained by intellectual deterioration, poor comprehension, uncooperativeness or a deficit in the elemental motor or sensory system [9]. Frequently observed clinical symptoms of LA are an inability to perform purposeful movements with one’s arms or hands, errors when asked to demonstrate how to use an object or how to carry out actions involving a single or series of components of movements, and problems imitating abstract and symbolic gestures [5, 10]. Thus, with LA, performance is characterized by a series of errors leading to an incomplete, inaccurate or incorrect gesture [5, 10].

Limb apraxia subtypes are based on the classification of Hugo Liepmann [11]. The subtypes of this syndrome are usually multimodal because they do not depend on the modality of perceptual visual, verbal, or tactile stimuli that the person receives [12]. The two subtypes of apraxia that have been described in the most detail in scientific literature are ideational apraxia (IA) and ideomotor apraxia (IMA). IA occurs when patients have problems performing a sequence of actions requiring the use of various objects in the correct manner and order necessary to complete an intended goal. IMA is the inability to properly perform gesture pantomimes and imitations, whereas the use of real tools is less affected. Patients that present with IMA know cognitively what to do but do not know how to execute the movement. The idea or plan for the action is not damaged, but implementation of the motor plan for turning gestures into actions is impaired [12].

The deficits linked to apraxia are typically associated with brain damage of vascular etiology, especially after left hemispheric stroke [13–15]. Studies report prevalence rates varying from 10 % to 50 % for the traditional clinical classification of IA and IMA deficits after lesions in the left parietal and premotor cortical areas [6, 14, 15]. Patients with right-brain damage and IMA have also been reported, with prevalence rates from 20 % to 54 % [16, 17].

Apraxia is therefore one of the common cognitive deficits that occur after a stroke. It can have negative impacts on a patient’s independence in activities of daily living (ADLs) [14] due to reduced levels of patient autonomy [18–20]. The disorder not only appears in clinical settings but also in many natural, day-to-day environments [10] where patients commonly perform ADLs, that is, the daily activities required to live safely and independently at home. The ecological relevance of apraxia has been reported in the ability of patients to perform various ADLs, for example, feeding, bathing, toileting, and grooming, as well as dressing and brushing one’s teeth. Moreover, gesture deficits impact negatively on patients’ nonverbal communication and the quality of communicative gestures. For this reason, patients who have apraxia rarely use spontaneous communicative gestures in daily living settings [5, 19–24].

To promote the independence and safety of apraxia patients’ daily functional performance, efficient cost-effective and evidence-based intervention strategies for LA are needed. The most important interventions reported in the scientific literature that are currently used for upper limb apraxia can be divided into two approaches: restorative and compensatory. With the restorative method, damaged systems/processes are treated directly, the objective being to bring the partially affected abilities back to their functional level prior to illness. It focuses on the training of transitive and intransitive gestures, with the aim of restoring the patient’s pre-morbid level of function. ADL training is most successful where patients have practiced the activities during their daily routines in natural everyday environments. This gesture execution treatment regimen was developed by Smania and collaborators [6, 10, 25].

Compensatory methods are based on providing strategies for patients to compensate for deficits associated with apraxia. By definition, a compensatory treatment does not lead to the recovery of specific abilities but requires the damaged system to generate compensatory mechanisms. This approach was developed, with specific training strategies, by the Dutch group of Van Heugten and collaborators [6, 12, 26, 27].

Previous studies have reported that rehabilitative treatment of the cognitive functioning of patients with LA after a stroke can bring about significant improvements in the performance and recognition of both transitive and intransitive gestures. Regaining praxis skills often generalizes to improvements in ADL functioning as well. Furthermore, follow-up evaluations indicate that it could have long-term treatment benefits [10, 25].

Evidence of the effectiveness of compensatory strategies through ADL training has also been found in this client group. Results after an eight-week treatment period indicate that integrating specific strategy training into the usual occupational therapy was more effective in improving ADL functioning than usual occupational therapy alone [14]. The results of a similar strategy training approach demonstrated clinically large and statistically significant impacts on all measures of ADL functioning, although small significant improvements were found in tests on apraxia and motor functioning. In the same study, 71 % of the patients had improved Barthel Index scores. These results suggest that the training appears to be successful in teaching patients compensatory strategies that enable them to function more independently, despite the residual impacts of apraxia [26]. However, as this study did not include a control group, these findings could have been affected by other factors. Although previous research has shown that the effective management and treatment of LA has the potential to have a marked impact on patient outcomes, more rigorous empirical evidence is needed to demonstrate the effectiveness of rehabilitation approaches for upper limb apraxia [10]. Only a few studies have been published that have a suitably rigorous study design and a sufficiently large sample size. Also, the efficacy of different apraxia treatments has only been investigated in a few randomized controlled studies [5].

There is some debate as to whether teaching compensatory strategies reduces the likelihood that restorative approaches will be successful because, once learned, compensation is very difficult to modify; that is, it may be difficult to unlearn compensated movement, and this may frustrate any efforts to improve movement with restorative strategies [28, 29]. However, other authors claim that teaching compensatory and restorative frameworks should be combined to determine whether this is in fact true or whether, on the contrary, greater positive effects are achieved with a combined approach.

To our knowledge, no studies have been completed using a combined intervention approach, where patients receive cognitive training on apraxia in conjunction with a method that focuses on the strategies needed to promote ADL functional performance. Moreover, we are not aware of any studies that incorporate an apraxia intervention at home, in other words, in a natural day-to-day context. Hence, a more rigorously designed large scale randomized trial is needed to evaluate the effectiveness of a combined treatment approach to functional rehabilitation at home that integrates both intervention approaches to LA function (restorative and compensatory) with autonomy in ADLs and quality of life (QoL) in poststroke patients.

For these reasons, the overall objective of this randomized control trial is to evaluate the immediate and medium-term effects of a combined functional rehabilitation intervention aimed at improving mild to moderate upper limb apraxia in poststroke patients, using a combination of restorative and compensatory approaches in the patients’ home environments, in comparison with a control group who undergo a traditional health education protocol. The specific research aims are (a) to investigate the impact of a combined functional rehabilitation intervention approach on the independence of patients presenting with an upper limb apraxia that affects the performance of their basic/instrumental activities of daily living, (b) to compare the neuropsychological functional status of patients’ LA, and (c) to assess the impact of this combined intervention in upper limb apraxia on the patient’s quality of life.

Our study hypothesis is that changes induced by the restorative approach in combination with the compensatory approach could have positive effects on the performance of transitive/intransitive gestures, ADLs and QoL in patients with upper limb apraxia.

## Methods/Design

### Research design

This study will be conducted as a two-arm patient and therapist-blinded, parallel, randomized controlled trial design, following the recommendations of the SPIRIT guidelines (Additional file 1) [30]. The experimental group will receive a combined functional rehabilitation program at home and will be compared to a control group receiving a conventional health education program. The main outcome measures will be collected at three time points over a 16-week period: (1) at baseline, (2) at posttreatment (that is, 8 weeks after baseline), and (3) at follow-up (16 weeks after baseline). The study assessment schedule is shown in Table 1. The study was approved on 26 May 2014 by the Investigation Ethics Committee of Granada province-CEI (Andalusian Health Service, Granada, Spain, 180SP) and complies with the 2013 modification of the Helsinki Declaration [31] and with current Spanish legislation for clinical trials [32].

**Table 1:** Schedule of enrollment, interventions, and assessments of the study

	Enrollment	Allocation	Post-allocation		
Time point	Pre-treatment	Time 0	Baseline	8-weeks posttreatment	2-month follow-up
Enrollment:					
Eligibility screen	X				
Informed consent	X				
Allocation		X			
Interventions:					
Combined functional rehabilitation of apraxia group			X	X	
Traditional health educative protocol group			X	X	
Assessments:					
Sociodemographic and clinical data			X		
Barthel index			X	X	X
Instrumental Activities of Daily Living Scale			X	X	X
Observation and scoring of ADL-Activities			X	X	X
De Renzi Test for ideational apraxia			X	X	X
De Renzi Test for ideomotor apraxia			X	X	X
Recognition of gestures			X	X	X
Test of upper limb apraxia (TULIA)			X	X	X
Quality of life scale for stroke (ECVI-38)			X	X	X

### Participants

**Fig. 1:**
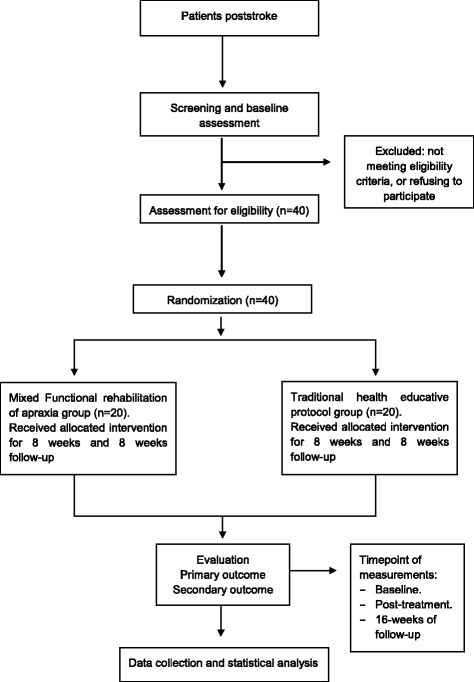
Flow diagram of study design

Forty patients with clinical evidence of left and right-sided unilateral vascular lesions poststroke and upper limb apraxia will be recruited from the Neurology Unit of San Cecilio Hospital in Granada, Spain. Initial contact with the patients will be made by telephone 2 months after they sustained the stroke. Participants will be asked to talk face-to-face with the coordinators to discuss the study and obtain information regarding the objectives and characteristics of the research. Those who give their voluntary consent for participating in the study will be interviewed and assessed by a neurologist to determine whether they meet the study inclusion criteria. Written informed consent will then be obtained from all those taking part in the study. Figure 1 shows the flow diagram of the study participants.

### Eligibility criteria

The criteria for inclusion in the study will be as follows: (a) age between 25 and 95 years; (b) presenting with mild to moderate effects of a stroke 2 months after a cerebrovascular attack (this will be determined from a neurological examination and completion of the NIH stroke scale [33, 34]; (c) the presence of upper limb apraxia lasting at least 2 months; (d) voluntary participation; (e) intervention judged to be necessary by the neurologist, occupational therapist. and patient; and (f) patients presenting with apraxia, determined by a score of less than or equal to nine points on the validated tool for apraxia screening (Apraxia screen TULIA) [35].

The exclusion criteria have been established as follows: (a) a history of apraxia predating the current stroke; (b) having had a stroke less than 2 months or more than 24 months previously; (c) cognitive impairment (<23 points in the normal school population and <20 points with a low level of education or illiteracy, in accordance with the Spanish version of the Mini-Mental State Examination) [36]; (d) severe aphasia (when the patients do not understand the commands or are unable to complete the screening tests); (e) a previous brain tumor; (f) a previous history of other neurological disorders; (g) having a mother tongue other than Spanish; (h) a history of known drug addiction; (i) a known intellectual or learning disorder; (j) brain damage from a traumatic or neurodegenerative process (k) a history of serious impairment of awareness; (l) the presence of orthopedic or other disabling conditions; and (m) the patient withholding consent to take part in the study.

### Randomization and blinding

The patients who volunteer to take part in the study will be randomly allocated into two groups (the experimental and control groups), using a computational random number generator (EPIDAT 3.1, Xunta de Galicia) with randomization codes created by MCGR. This will assign patients to a combined functional apraxia rehabilitation group or a control group with an allocation ratio (1:1). During the randomization process, patients will be blinded to their treatment allocation. The neurologist will examine the patients for eligibility criteria and collect all the baseline demographic variables but will not be involved in the rest of the study or preparation of the randomization codes. Treatment allocation will be concealed and patients and study personnel will be blinded to the treatment assignments until after the database has been locked. During the trial, all the outcome measures will be collected by three therapists, who will be blinded to the group allocation. Treatment interventions will all be carried out by an occupational therapist with wide clinical experience, who will be blinded to the outcome measures and baseline examination findings but not to the patients’ treatment allocation, although this allocation will not be revealed to the therapists who gather the outcome measure data. Criteria for receiving discontinued intervention will be as follows: 1) the participant requests voluntarily to miss any session, or 2) the interventions produce a significant improvement or worsening of apraxia disease. As part of the strategy to improve adherence to intervention, patients will be informed about the relevance of the treatment in each session, and they will be telephoned weekly to remind the date of the sessions. The final result of the study will be reported to the patients in both groups.

### Combined functional rehabilitation approach to apraxia (experimental group)

The functional rehabilitation approach for upper limb apraxia consists of occupational therapy intervention based on two complementary approaches. Information about these approaches has been published separately, and there is evidence that both approaches are effective [6, 10, 26]. The experimental group will receive an 8-week combined functional rehabilitation program at home with an occupational therapist on 3 days per week in 30-min intervention periods, focusing on restorative and compensatory techniques for upper limb apraxia. Once the occupational therapist has identified the patient’s needs, the restorative program will be used in the first two of the weekly sessions, and the compensatory methods in the last session of the week. Both methods will be used to improve the patients’ functional performance, allowing them to be more independent in different daily living contexts, especially in the home.

In the restorative approach, developed by Smania et al. [6, 10], treatment is designed in three sections, directed towards transitive, intransitive-symbolic and intransitive-nonsymbolic gestures, respectively. Transitive gesture training is subdivided into three phases: A, B, and C. In phase A, the patient is required to demonstrate the use of common tools. In phase B, the patient is shown a picture illustrating a transitive gesture and is then required to mime or imitate that gesture. In phase C, the patient is presented with a picture showing a common tool and is then asked to demonstrate how it is used by pantomiming. Each phase contains 20 items. When the patient is able to correctly perform at least 17 of the 20 items, that phase is concluded and the following phase is initiated [6, 10].

Intransitive-symbolic gesture training is also subdivided into phases A, B, and C, depending on the number of contextual cues used under the different conditions. In intransitive-nonsymbolic gesture training, the patient is asked to imitate meaningless intransitive gestures previously shown by the examiner. Twelve gestures, involving six proximal and six distal joints, are delivered. Half of the gestures are static, and the rest are dynamic. Patients who are unable to perform the gesture properly are assisted by the examiner with verbal or other appropriate facilitation [6, 10].

The compensatory approach is based on the work of Van Heugten et al. [6, 26]. The treatment consists of therapy sessions that focus on teaching patients strategies to compensate for deficits linked to apraxia. Greater improvement in ADL functioning is typically expected from implementation of the compensatory approach, rather than the recovery of motor impairments. Patients become more independent in performing everyday activities when they are taught internal and external strategies, such as the use of oral and written instructions, or picture sequences that can compensate for any functional deficits caused by apraxia during the execution of their everyday activities. Compensation can be provided in two ways: externally (by the therapist), using materials that help the patient to perform an action (for example, photographs depicting the correct sequence of an action), and internally (generated by the patients themselves) by relying on intact cognitive functions (for example, verbalizing the sequence of distinct actions the patient is supposed to complete) [6, 26].

The decision about which activity to work on will be made collaboratively with the patient and the occupational therapist. The therapist will take into account the decision tree and principles used by Van Heugten et al. to guide the selection of a specific activity and apply a checklist of activities. The checklist will consist of an assessment of the activities the patient performed prior to the stroke and those activities that are relevant to the patient for future performance. The interventions implemented during the compensatory sessions are based on specific difficulties noted during standardized ADL observations [6, 26].

ADL activities are conceived as three successive phases, conforming to the information processing framework: initiation, execution, and control. For example, a patient with apraxia who cannot use objects appropriately may have a deficit at any one of the stages of the activity being performed. By evaluating the different components of the activity, the structure of the deficit can be recognized, and the design of the treatment can be formulated accordingly [6, 26].

### Traditional health education protocol (control group)

The control group patients will receive a health education program only. They and their caregivers will attend a series of education workshops where they will be given information about what having a stroke involves, the meaning of upper limb apraxia, the implications of upper limb apraxia on daily living at home, and the common types of challenges arising from apraxia with respect to the daily use of a person’s upper limb. The workshops will take place at the patient’s home once a month over a 2-month period. After completion of the study, this group will be offered the opportunity to participate in the combined approach intervention, although the data from this will not be used in the statistical analysis.

### Assessment

#### Primary outcome measure

##### Barthel Index

The Barthel Index (BI) for ADLs is a widely used standardized scale for assessing functional disability in basic ADLs. It scores ten basic activities: bowel control, bladder control, grooming, toilet use, feeding, transfer, mobility, dressing, using the stairs and bathing. The score generated varies from zero (totally dependent) to a maximum score of 20 (totally independent). It has good interobserver reliability, with a Kappa statistic between 0.47 and 1.00, and good intra-observer reliability, with a Kappa statistic between 0.84 and 0.97 [37]. In a study involving a poststroke participant group, the internal consistency of the BI was a Cronbach’s alpha of 0.92 [38].

### Secondary outcome measures

#### General functionality and autonomy

The Lawton and Brody Instrumental Activities of Daily Living Scale is a questionnaire used to assess the ability to develop the instrumental activities necessary for living independently in the community. It is a self-administered scale validated for the Spanish population [39]. The maximum score of 8 points indicates complete independence, a score of 6 to 7 mild dependence, 4 to 5 moderate dependence, 2 to 3 severe dependence, and 0 to 1 total dependence. It has high inter- and intra-observer coefficients of reliability (0.94) and high test-retest reliability (0.95) [39, 40].

The Observation and Scoring of ADL Activities is a test that consists of observing the performance of activities of daily living with a system of standardized observations specially developed for assessing disability caused by apraxia. The overall score varies from totally dependent (0) to fully independent (3). The Kappa statistic shows values higher than 0.70, indicating considerable agreement [38]. The inter-observer reliability of each assessed activity varies with the intraclass correlation coefficients from 0.62 to 0.98 [41].

#### Neuropsychological tests

The De Renzi Test for Ideational Apraxia assesses ideational apraxia in the upper limbs and requires the use of real objects. Patients are asked with words and gestures to take an object in their hands and demonstrate how they would use it. Two points are assigned for an immediate correct response, and one point is assigned if a correct performance is preceded by hesitation or a protracted latency period during which wrong or unsuccessful movements are presented or if the performance is conceptually correct but the actual movements are somewhat inaccurate or awkward. For any other type of error, no points are given. The score varies from zero to 14 points [16].

The De Renzi Test for Ideomotor Apraxia requires patients to reproduce a wide variety of intransitive gestures, that is, gestures that do not require the use of objects. The gestures may be symbolic (for example, the OK sign) or nonsymbolic (hand under the chin). Patients are assigned a maximum of three points if they perform the gestures correctly after the first demonstration, and two or one points, depending on whether they need a second or third demonstration. If all the demonstrations are unsatisfactory, no points are given. The test consists of 24 items and the total test score varies from zero to 72 points [42].

Recognition of Gestures tests both transitive and intransitive-symbolic gestures, following the recommendations of Smania et al. [10]. For transitive gestures, the patient is given three pictures showing an action performed a) with an object, b) with a semantically related but inappropriate object, or c) with a semantically unrelated inappropriate object. Patients are required to indicate the picture in which the correct transitive gesture is reproduced. As for intransitive symbolic gestures, the patient is given three pictures showing different symbolic gestures, one of which is related to a context represented in another picture. The remaining two pictures show gestures with or without postural affinities with the correct gesture. The patient is requested to indicate the picture showing the gesture related to the context. The test includes five transitive and five intransitive gesture recognition trials. One point is given for each correct response, with the resultant scores varying from zero to ten points [10].

The Test of Upper Limb Apraxia (TULIA) for Comprehensive Assessment of Gesture Production has a total of 48 items, grouped into six subtests, for imitating gestures and simulating gestures that are nonsymbolic (meaningless), intransitive (communicative), and transitive (related-objects). It has a Likert scale from zero to five points per item, with a total score varying from zero to 240 points [43]. Evidence shows that this test is a reliable and valid assessment of gesture production. It can therefore be easily applied for the purposes of research and clinical practice. It has a good to excellent internal consistency and test-retest reliability at both the level of the six subtests and the individual level of the items. For test-retest reliability at item level in patients examined three times within 24 hours, it has been shown that most items (*n* = 39) had a good-to-perfect degree of agreement (varying from 0.66 to 1.0) [43].

#### Overall quality of life

The Quality of Life Scale for Stroke (ECVI-38) is a self-administered questionnaire, but in cases where the patient is unable to read or understand the questions, it can be completed by a relative or the primary caregiver. It has 38 items grouped into eight subscales: physical, communication, cognition, emotions, feelings, basic activities of daily living, common activities of daily living, and social and family functioning, plus two additional questions about involvement in sexual relationships and occupational activity. Responses are categorized depending on the percentage obtained of the total maximum score of 100 points. Less than 25 % means unaffected, between 25 and 50 % indicates mild disease, between 50 and 75 % shows moderate impairment, and 75 % or more means severely affected. This test showed a test-retest reliability varying from 0.81 to 0.96, calculated with intraclass correlation coefficients examined at 1 and 2 weeks [44, 45].

##### Incidence of adverse events

Although no previous adverse effects have been reported for this intervention, participants will be asked to provide information about any potential adverse events. These will be recorded by the researcher during the trial and follow-up. Adverse events might include upper limb pain an hour after treatment, musculoskeletal impairments, and swelling or increased muscle tone in the upper limb. The researcher will collect data on these events, such as time and date of occurrence, severity, measurements related to the treatment and causal relationship with the intervention. Severe adverse events will be reported immediately to the principal investigator (MEAF).

### Sample size

Based on previously published findings on the use of rehabilitation treatments by poststroke patients with upper limb apraxia [14], a clinically important difference pre-post treatment of 2.44 points on the Barthel Index for ADLs (primary outcome) was used to calculate the sample size required to detect the increase in patients’ functional ability with combined functional rehabilitation (experimental group) versus the traditional health educative protocol (control group), using the PASS 13 (Power Analysis and Sample Size) program (http://www.ncss.com/). A sample size of 15 patients per arm was estimated to provide a 95 % confidence interval (CI) with a power of 80 %, assuming a standard deviation of 3.10 points for this difference and a two-sided test (α) of 0.05. The sample size has been increased to a total of 40 to allow for a loss to follow-up of up to 22 %.

### Statistical analysis

The data obtained in this study will be analyzed with SPSS software, version 20.0. The reliability of the model and validity of the hypothesis will be checked and the normal distribution of variables, residuals values and homogeneity of the variances will be studied. The normal distribution of variables will be verified by the Kolgomorov-Smirnov test for continuous variables and the Chi-Square Goodness of Fit test for categorical variables. Residuals will be analyzed using the residuals graph to compare observed values against the residuals values, distribution and tendency. Linearity will be examined using bivariate scatter plots of observed residual values against expected values.

Descriptive statistics will be used first to characterize the study group. The outcomes will be reported with the mean and standard deviation of scores at baseline, post-treatment and follow-up time. To evaluate the main effect of the independent variable (interventions) on outcomes and potential time-by-treatment interactions, repeated measures analysis of variance (RM ANOVA) will be conducted. To check for sphericity, the variances of the differences between all combinations of groups of within-subjects factors will be analyzed with the Mauchly test and verified by the local invariant test. Although the Mauchly test is the most widely used, both tests will be performed because some authors have reported that the local invariant test produces fewer Type II errors than the Mauchly test with small sample sizes and a large number of variables. The Levene test will be used to assess the equality of variances at the 0.05 significance level. Post hoc analyses of significant group x time interaction effects will be carried out using the Tukey multiple comparison procedure to examine pairwise comparisons of the groups’ means for all outcome measures and across the three data collection times.

To complete the analysis, the effect size and level of significance for interacting effects (group × time) will be shown, using Cohen’s d (standardized difference between two means). The interpretation of effect size will be made following the guidelines of ≥ 0.2 as small effect, ≥ 0.5 as medium effect, and 0.8 and above as large effect, in accordance with Cohen. If normality and homoscedasticity assumptions are violated, data transformation and non-parametric procedures will be considered. Data from drop-outs and patients who discontinued intervention will be analyzed following an intention-to-treat principle. A P value of 0.05 will be used for statistical significance.

## Discussion

This study on the functional rehabilitation of upper limb apraxia will analyze the effects on post-stroke patients of an 8-week integrative and innovative intervention program, focused on two different but complementary approaches for improving the functional performance, autonomy and cognitive function of patients presenting with apraxia. The intervention will take place in the context of the patients’ homes, which provide a natural day-to-day living environment for the study participants.

Apraxia rehabilitation has been researched and reported on in the peer-reviewed literature [2, 5, 6, 46], especially with respect to stroke patients [6, 14], and has been shown to be effective in clinical settings immediately after treatment and on for a longer term. LA has an irrefutable impact on patients’ ADL independence. Although spontaneous recovery of upper limb apraxia is common, it typically does not occur in apraxia patients with ADL abilities; hence, an adequate training can enhance independence and accelerate the natural recovery process [19]. Compensatory and restorative approaches have independently been shown to be effective in improving patients’ ADL function [10, 19, 26, 47]. Both frameworks can be joined together to combine the single effects of each approach, resulting in an effective recovery of the patients. In other words, it is expected that a combination of both approaches will produce better functional outcomes and a better overall patient QoL.

### Limitations

The first limitation involves the methodology. If it were feasible, a three-arm study including a restorative, a compensatory and a combined intervention group might provide greater insights. However, we have designed a study for investigation purposes in which a combined intervention is compared with a control group, since we could find no previous studies that have used this intervention approach with apraxia disorders.

Since the two groups receive different amounts of intervention, there is also potential for attention bias. However, the authors are required to comply with the protocol for upper limb apraxia at the hospital from which the patients are recruited, and it is not possible to change the control group.

The second limitation is that not all the original validation articles of the measures used in the present study, such as the De Renzi test for ideomotor apraxia, the recognition of gestures tests and the Barthel test, reported test-retest reliability. This is a critical aspect when potential clinical changes over time are being measured. Despite this, we have opted to use these tools because of the frequency with which they have been utilized in previous apraxia research.

The third limitation is that we could find no previous reports that show the effect size of the combined intervention; therefore, our sample size may not be adequate. However, in a clinical context, we hope that the combined approach achieves at least the minimum clinically important difference shown for other apraxia treatments. It is for this reason that the sample size has been calculated based on this value. In addition, recruiting patients with upper limb apraxia is difficult. As a result, previous research on this issue has used a smaller or similar sample size.

## Trial status

Participant enrollment began on 1 May 2014. The trial is expected to be completed in March 2016.

## Additional files

Additional file 1:
**SPIRIT checklist.** Fulfilled *SPIRIT* (Standard Protocol Items: Recommendations for Interventional Trials) checklist of the clinical trial protocol for functional rehabilitation of upper limb apraxia. (PDF 116 kb)

## Abbreviations used

ADLs: independence in activities of daily living
BI: Barthel Index
LA: limb apraxia

## References

[1] Gross RG, Grossman M (2008). Update on apraxia. Curr Neurol Neurosci Rep.

[2] Buxbaum LJ, Haal KY, Hallett M, Wheaton L, Heilman KM, Rodriguez A (2008). Treatment of limb apraxia: moving forward to improved action. Am J Phys Med Rehabil.

[3] Tirapu-Ustárroz J, Ríos-Lago M, Maestú-Unturbe F (2008). [Neuropsychology manual].

[4] Ochipa C, Rothi LJG (2000). Limb apraxia. Semin Neurol.

[5] Dovern A, Fink GR, Weiss PH (2012). Diagnosis and treatment of upper limb apraxia. J Neurol.

[6] Cantagallo A, Maini M, Rumiati RI (2012). The cognitive rehabilitation of limb apraxia in patients with stroke. Neuropsychol Rehabil.

[7] Rumiati RI, Papeo L, Corradi-Dell’Acqua C (2010). Higher-level motor processes. Ann N Y Acad Sci.

[8] Leiguarda RC, Marsden CD (2000). Limb apraxias, higher-order disorders of sensorimotor integration. Brain.

[9] Heilman KM, Rothi LJG, Heilman KH, Valenstein E (1993). Apraxia. Clinical neuropsychology.

[10] Smania N, Girardi F, Domenicali C, Lora E, Aglioti S (2000). The rehabilitation of limb apraxia: a study in left-brain-damaged patients. Arch Phys Med Rehabil.

[11] Goldenberg G (2003). Apraxia and beyond: life and work of Hugo Liepmann. Cortex.

[12] Vanbellingen T, Bohlhalter S (2011). Apraxia in neurorehabilitation: classification, assessment and treatment. NeuroRehabilitation.

[13] Zwinkels A, Geusgens C, van de Sande P, Van Heugten C (2004). Assessment of apraxia: inter-rater reliability of a new apraxia test, association of apraxia and other cognitive deficits and prevalence of apraxia in a rehabilitation setting. Clin Rehabil.

[14] Donkervoort M, Dekker J, Stehmann-Saris FC, Deelman BG (2001). Efficacy of strategy training in left hemisphere stroke patients with apraxia: a randomised clinical trial. Neuropsychol Rehabil.

[15] Donkervoort M, Dekker J, Deelman B (2006). The course of apraxia and ADL functioning in left hemisphere stroke patients treated in rehabilitation centres and nursing homes. Clin Rehabil.

[16] De Renzi E, Motti F, Nichelli P (1980). Imitating gestures. A quantitative approach to ideomotor apraxia. Arch Neurol.

[17] Kaya K, Unsal-Delialioglu S, Kurt M, Altinok N, Oael S (2006). Evaluation of ideomotor apraxia in patients with stroke: a study of reliability and validity. J Rehabil Med.

[18] Hanna-Pladdy B, Heilman KM, Foundas AL (2003). Ecological implications of ideomotor apraxia: evidence from physical activities of daily living. Neurol.

[19] Goldenberg G, Hagmann S (1998). Therapy of activities of daily living in patients with apraxia. Neuropsychol Rehabil.

[20] Sundet K, Finset A, Reinvang I (1988). Neuropsychological predictors in stroke rehabilitation. J Clin Exp Neuropsychol.

[21] Foundas AL, Macauley BL, Raymer AM, Maher LM, Heilman KM, Gonzalez Rothi LJ (1995). Ecological implications of limb apraxia: evidence from mealtime behavior. J Int Neuropsychol Soc.

[22] Hermsdorfer J, Hentze S, Goldenberg G (2006). Spatial and kinematic features of apraxic movement depend on the mode of execution. Neuropsychology.

[23] Feyereisen P, Barter D, Goossens M, Clerebraut N (1988). Gestures and speech in referential communication by aphasic subjects: channel use and efficiency. Aphasiology.

[24] Borod JC, Fitzpatrick PM, Helm-Estabrooks N, Goodglass H (1989). The relationship between limb apraxia and the spontaneous use of communicative gesture in aphasia. Brain Cogn.

[25] Smania N, Aglioti SM, Girardi F, Tinazzi M, Fiaschi A, Cosentino A (2006). Rehabilitation of limb apraxia improves daily life activities in patients with stroke. Neurology.

[26] Van Heugten C (1998). Outcome of strategy training in stroke patients with apraxia: a phase II study. Clin Rehabil.

[27] Van Heugten J, Dekker BG (2000). Rehabilitation of stroke patients with apraxia: the role of additional cognitive and motor impairments. Disabil Rehabil.

[28] Jeyaraman S, Kathiresan G, Gopalsamy K (2010). Normalizing the arm reaching patterns after stroke through forced use therapy–a systematic review. Neurosci Med.

[29] Giles GM, Clark-Wilson J (1999). Rehabilitation of the severely brain-injured adult: a practical approach.

[30] Chan AW, Tetzlaff JM, Gøtzsche PC, Altman DG, Mann H, Berlin J (2013). SPIRIT 2013 explanation and elaboration: guidance for protocols of clinical trials. BMJ.

[31] World Medical Association (2013). World Medical Association Declaration of Helsinki: ethical principles for medical research involving human subjects. JAMA J Am Med Assoc.

[32] [Law of Biomedical research]. Law 14/2007 of 3 July. Boletin Oficial del Estado, n° 156, (04-05-2007). (Spanish).

[33] Montaner J, Alvarez-Sabín J (2006). [NIH stroke scale and its adaptation to Spanish]. Neurologia.

[34] Domínguez R, Vila JF, Augustovski F, Irazola V, Castillo PR, Escalante RR (2006). Spanish Cross-Cultural Adaptation and Validation of the National Institutes of Health Stroke Scale. Mayo Clin Proc.

[35] Vanbellingen T, Kersten B, Van de Winckel A, Bellion M, Baronti F, Müri R (2011). A new bedside test of gestures in stroke: the apraxia screen of TULIA (AST). J Neurol Neurosurg Psychiatry.

[36] Lobo A, Ezquerra J, Gómez-Burgada F, Sala JM (1979). [Cognitive mini-test: a simple practical test to detect intellectual changes in medical patients]. Actas Luso Esp Neurol Psiquiatr.

[37] Cid-Ruzafa J, Damián-Moreno J. [Evaluating physical incapacity: the Barthel Index]. Rev Esp Salud Pública. 1997; 71:127–37 (Spanish). 9546856

[38] Van Heugten CM, Dekker J, Deelman BG, van Dijk AJ, Stehmann-Saris FC, Kinebanian A (2000). Measuring disabilities in stroke patients with apraxia: a validation study of an observational method. Neuropsychol Rehabil.

[39] Olazarán J, Mouronte P, Bermejo F (2005). [Clinical validity of two scales of instrumental activities in Alzheimer’s disease]. Neurologia.

[40] Lawton MP, Brody EM (1969). Assessment of older people: self-maintaining and instrumental activities of daily living. Gerontologist.

[41] Van Heugten CM, Dekker J, Deelman BG, Stehmann-Saris JC, Kinebanian A (1999). Assessment of disabilities in stroke patients with apraxia: internal consistency and inter-observer reliability. Occup Ther J Res.

[42] De Renzi E, Pieczuro A, Vignolo L (1968). Ideational apraxia: a quantitative study. Neuropsychologia.

[43] Vanbellingen T, Kersten B, Van Hemelrijk B, Van de Winckel A, Bertschi M, Müri R (2010). Comprehensive assessment of gesture production: a new test of upper limb apraxia (TULIA). Eur J Neurol.

[44] Fernández-Concepción O, Ramírez-Pérez E, Álvarez MA, Buergo-Zuazbar MA (2008). [Validation of the stroke-specific quality of life scale–ECVI-38]. Rev Neurol.

[45] Soriano-Guillén AP, Coarasa-Lirón-de-Robles A, Reigada-Pérez-de-Santa-Cruz P, Solano-Bernad V (2013). [Use of the stroke-specific quality of life scale (ECVI-38) to quantify and measure the consequences of a stroke. Relation with demographic and clinical variables]. Rehabilitación.

[46] Landry J, Spaulding S (1999). Assessment and intervention with clients with apraxia: contributions from the literature. Can J Occup Ther.

[47] Goldenberg G, Daumüller M, Hagmann S (2001). Assessment and therapy of complex activities of daily living in apraxia. Neuropsychol Rehabil.

